# Society for cardiovascular magnetic resonance recommendations for training and competency of CMR technologists

**DOI:** 10.1186/s12968-022-00900-1

**Published:** 2022-12-05

**Authors:** Stephen Darty, Elizabeth Jenista, Raymond J. Kim, Christopher Dyke, Orlando P. Simonetti, Monika Radike, Jen Bryant, Chris Benny Lawton, Nicole Freitag, Dipan J. Shah, Chiara Bucciarelli-Ducci, Subha Raman, Sven Plein, Michael D. Elliott

**Affiliations:** 1 Division of Cardiology, Duke University, Durham, NC USA; 2 Division of Cardiology, National Jewish Health, Denver, CO USA; 3 Division of Cardiology, The Ohio State University, Columbus, OH USA; 4 Department of Radiology, Liverpool Heart and Chest NHS Foundation Trust, Liverpool, UK; 5 National Heart Centre, Singapore, Singapore; 6 St. Josephs Hospital, Newport, Wales UK; 7 Division of Cardiology, Allina Health, Minneapolis, MN USA; 8 Division of Cardiology, Houston Methodist Debakey Heart & Vascular Center, Houston, TX USA; 9 Division of Cardiology, Royal Brompton and Harefield NHS Foundation Trust, London, UK; 10 Division of Cardiology, Indiana University, Indianapolis, IN USA; 11 Division of Cardiology, University of Leeds, Leeds, UK; 12 Atrium Health/Sanger Heart & Vascular Institute, Charlotte, NC USA

## Abstract

The Society for Cardiovascular Magnetic Resonance (SCMR) recommendations for training and competency of cardiovascular magnetic resonance (CMR) technologists document will define the knowledge, experiences and skills required for a technologist to be competent in CMR imaging. By providing a framework for CMR training and competency the overarching goal is to promote the performance of high-quality CMR and to foster the increased adoption of CMR into clinical care.

## Background

The combination of a rapid pace of imaging innovation and clinical data has positioned cardiovascular magnetic resonance (CMR) as a vital imaging modality in the evaluation and management of a wide range of cardiovascular diseases. In particular, CMR is widely regarded as the optimal imaging modality for the assessment of biventricular volumes, regional and global systolic function, myocardial viability, stress perfusion imaging, dilated and infiltrative cardiomyopathy, and congenital heart disease (CHD). However, despite its expanding and important role in cardiovascular care it is widely acknowledged that there are significant regional variations in CMR service delivery. For instance, in 2017 there were more CMR studies performed in London, United Kingdom than in the entire United States Medicare population [1]. The inequities in access to CMR imaging can be linked to multiple challenges in the initiation and development of a CMR program. Competent and well-trained CMR technologists are fundamental to the success of a CMR program, and the current limited availability of CMR technologists is a challenge for CMR adoption more widely into clinical care.

Guidelines and/or formal education in CMR imaging for magnetic resonance (MR) technologists are neither widely available nor standardized worldwide. In the United States, MR technologists are typically educated in a 2-year Associate Degree program that includes MR physics, MR safety, instrumentation, patient care procedures, MR quality control and the performance of MR procedures. Notably, education and training in CMR is not required and often not included as an elective in MR technologist training programs. In fact, the American Registry of Radiologic Technologists (AART) the world’s largest organization offering credentials in MR and other medical imaging) does not recognize CMR as a separate MR procedure [2]. The ARRT requires demonstrated competence in 17 mandatory MR procedures and at least 11 of 30 elective MR procedures. CMR is not included as either a mandatory or elective MR procedure. Consequently, training in CMR is not a requirement to be a candidate for MR certification, nor is CMR content present in the ARRT certifying examination. In the United Kingdom, the standards of proficiency for radiographers from the Health and Care Professions Council does not have any requirements for content regarding CMR training [3].

Achieving proficiency in CMR imaging is time-intensive, requiring mastery of the application and adjustments of numerous cardiovascular pulse sequences and protocols. There are no shortcuts-CMR training is rigorous with a large time requirement needed to become an independent CMR technologist capable of consistently performing high quality CMR scans. Education in cardiovascular anatomy, physiology, pathology, and clinical indications for cardiac imaging is inherently necessary to achieve proficiency in CMR imaging, and are not a novel concept in technologist training. For example, an advanced level of cardiovascular education has been a long-standing component of the training of cardiac sonographers [4–6]. Furthermore, technologists receiving suboptimal CMR training can adversely impact patient care through the performance of low-quality, incomplete and longer duration CMR studies. The absence of formal CMR technologist training guidelines are a significant barrier to the successful implementation and expansion of CMR in clinical practice, and is problematic in several ways: (1) Without training standards, the pathway for technologists to achieve CMR proficiency is nebulous. (2) Determining the quality and/or comprehensive nature of a CMR training program without established guidelines is difficult for aspiring CMR technologists. Establishing CMR guidelines should foster the incorporation of education and training in CMR into MR technologist programs, facilitate the verification of technologist training and instigate pathways for healthcare systems to establish CMR technologist positions.

Despite the importance of the CMR technologist and the expanding role of CMR in cardiovascular care, there are no guideline recommendations for MR technologist training and competency in CMR. In recognition of the complex nature of CMR imaging and accelerating clinical demand for CMR, the Society for Cardiovascular Magnetic Resonance (SCMR) considers it a priority to provide guidelines for training and competency in CMR for MR technologists. A goal in establishing these guidelines is to address the limitations that exist for CMR technologist training and competency in order to facilitate the penetrance of CMR into clinical care. The SCMR has developed these guidelines to be broad-based in view of the marked heterogeneity of CMR centers worldwide. The objective of this report is to provide guidelines and competency milestones for MR technologists training in CMR. This document has been reviewed and approved by the SCMR Executive Committee.

## Recommendations for technologist training in CMR

This document defines the knowledge, experiences and skills required for a technologist to be competent in CMR imaging. By providing a framework for CMR training and competency the overarching goal is to promote the performance of high-quality CMR and to foster the increased adoption of CMR into clinical care.

The classification of knowledge and skills will be assigned into basic (Level 1), intermediate (Level 2), and advanced (Level 3) levels to mirror the categorization of CMR physician skill levels [7]. Complex CHD CMR, pediatric CMR, CMR under anesthesia and CMR post-processing are assigned to an additional Specialty CMR category. Estimates of the duration of training and number of CMR exams performed to achieve competency in the different levels is provided. The writing committee acknowledges that the time required to achieve proficiency in CMR imaging will vary reflective of diverse CMR practice settings, patient volumes and differing rates of skill acquisition amongst learners. As such, the number of cases performed and achievement of domain milestones is the determinant of a CMR technologist skill level rather than the cumulative training time needed to reach a skill level. The training and competency milestones for each skill level will be categorized into five domains: Patient safety/preparation, indications for CMR, electrocardiogram (ECG) gating, CMR scanning and CMR protocols [8].

The CMR technologist should achieve proficiency in each of the domains for a given skill level. The writing committee further acknowledges that the scope of clinical studies provided by CMR programs is varied. Therefore, the expectation is that a technologist demonstrate competency in at least 2/3 of the CMR protocols for each skill level. It should be emphasized that the CMR technologists must be capable of competently performing an extensive range of clinical CMR exams in order to meet Level 2 training requirements. Furthermore, technologists can perform Level 3 protocols even if they do not have the requisite training and/or experience to be a Level 3 CMR technologist. MR technologists who have completed Level 3 training will be qualified to serve in a supervisory and teaching role for a CMR program. The training supervision provided by a Level 2 or higher technologist or physician is to ensure that the training is consistent with site-specific protocols and the SCMR recommended protocols.

**Fig. 1:**
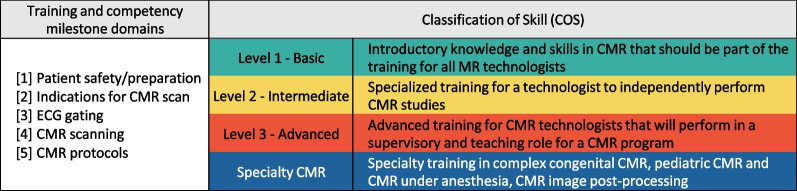
Classification of skill levels. *CMR* cardiovascular magnetic resonance, *ECG* electrocardiogram, *MR* magnetic resonance

**Fig. 2:**
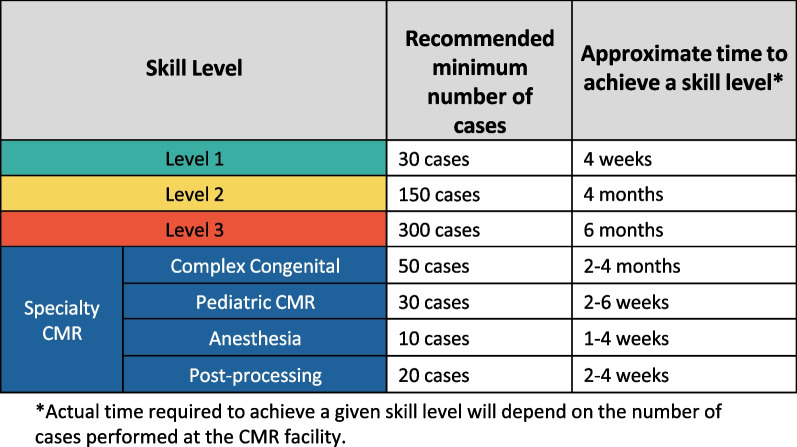
Minimum number of cases and expected time needed for each skill level. Note that only cases performed as the CMR operator should be counted towards competency

Importantly, the recommendations in this document should not be viewed or interpreted rigidly, as they are intended to guide the assessment of competency of a CMR technologist (Figs. 1, 2).

### Level 1 skills

**Fig. 3:**
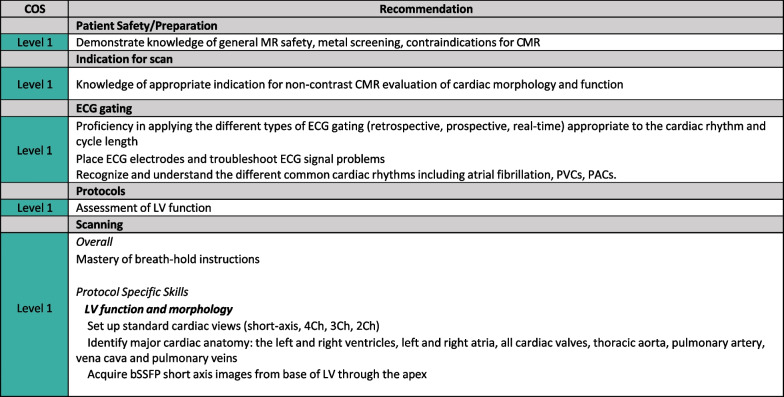
Level 1 skills. *2Ch* two-chamber, *3Ch* three-chamber, *4Ch* four-chamber, *bSSFP* balanced steady state free precession, *LV* left ventricle/left ventricular, *PACs* premature atrial contractions, *PVCs* premature ventricular contractions

These are the CMR foundational skills for a CMR technologist. Level 1 assumes general competency in the operation of a CMR scanner, as well as a prior degree or certification in MR. Level 1 competency encompasses a knowledge of the indications for the most common CMR procedures, and fluency in the technical considerations for acquiring cardiac images. This CMR training should be conducted under the supervision of a Level 2 or Level 3 (preferred) physician and/or technologist. A technologist with Level 1 skills can be expected to independently perform a basic exam for cardiac volumes and function. While a non-contrast CMR exam is limited in scope, it provides the technologist with the fundamental image acquisition skills and begins to familiarize the technologist with normal and abnormal cardiac findings. It is recommended that initial training should consist of observing at least 10 CMR studies. After the observation period, a minimum of 30 CMR exams is needed to acquire Level 1 CMR skills. Only cases performed as the primary scanner operator should be counted towards this competency. Although the length of time needed to achieve Level 1 skills may vary by center CMR volume, in general, it should take 4 weeks total (observation of 10 cases followed by the performance of 30 CMR exams) (Fig. 3).

### Level 2 skills

These skills encompass the requirements needed to perform CMR for the majority of clinical indications and build on the foundational Level 1 skills. Similar to Level 1, this CMR training should be conducted under the supervision of a Level 2 or Level 3 (preferred) physician and/or technologist.

**Fig. 4:**
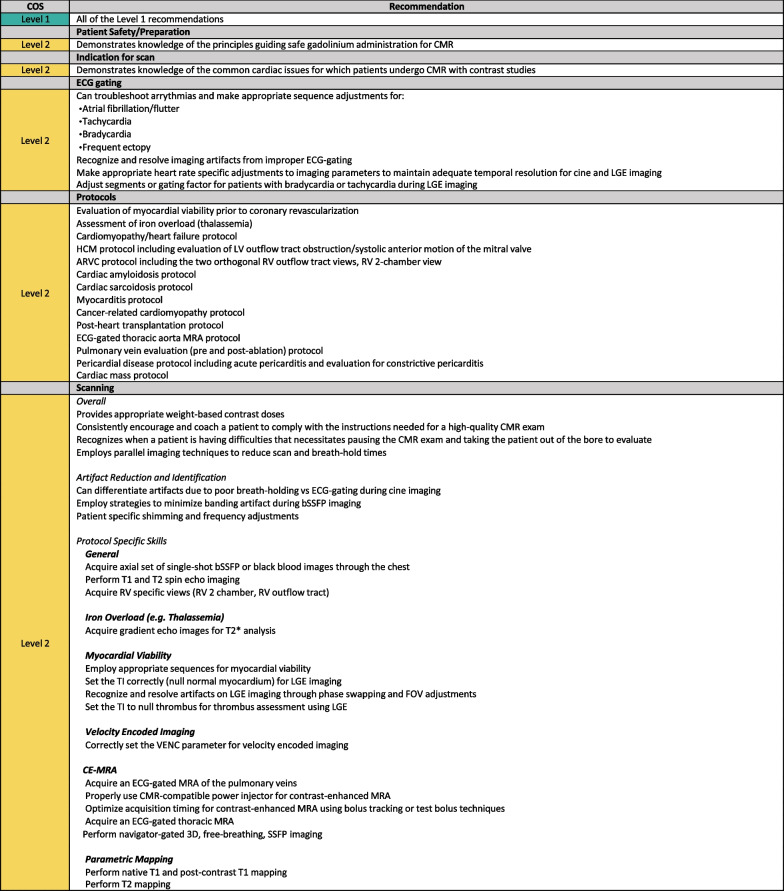
Level 2 skills. *ARVC* arrhythmic right ventricular cardiomyopathy, *HCM* hypertrophic cardiomyopathy, *LGE* late gadolinium enhancement, *MRA* magnetic resonance angiography, *RV* right ventricle/right ventricular, *TI* inversion time, *VENC* velocity encoding

A CMR technologist with Level 2 skills can be expected to perform post-contrast examinations, including myocardial viability, assessment of heart failure and infiltrative cardiomyopathies, hypertrophic cardiomyopathy, myocarditis, arrhythmogenic cardiomyopathy, pericardial disease and assessment of cardiac masses. As this level of competency is significantly broader than Level 1, it is recommended that performing at least 150 CMR exams is needed to acquire Level 2 CMR skills. Although the length of time needed to achieve Level 2 skills may vary by center CMR volume, 4 months of CMR imaging is recommended (Fig. 4).

### Level 3 skills

**Fig. 5:**
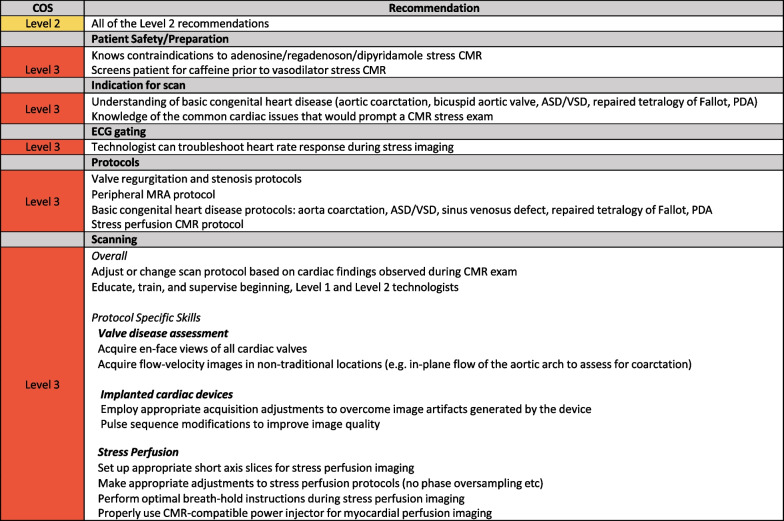
Level 3 skills. *ASD* atrial septal defect, *PDAi* patent ductus arteriosus, *VSD* ventricular septal defect

The skills that encompass Level 3 training are for CMR centers that perform less common, advanced CMR studies including assessment of valvular heart disease, basic CHD, CMR scanning of device patients, vasodilator stress perfusion imaging and advanced magnetic resonance angiography (MRA) techniques. This CMR training should be conducted under the supervision of a Level 3 physician and/or technologist. As these CMR scans are inherently more complex, the CMR technologist will need a more advanced knowledge of cardiac anatomy and physiology, in addition to possessing a solid understanding of the interplay of different CMR sequence parameters that govern temporal and spatial resolution, SNR, and scan duration. The CMR technologist with Level 3 skills should be able to adjust or change the scan protocol based on cardiac findings observed during the CMR exam. The Level 3 technologist should be able to provide preliminary findings of the CMR exam to the attending physician. It is recommended that at least 300 CMR exams are needed to acquire Level 3 CMR skills. Although the length of time needed to achieve Level 3 skills may vary by center CMR volume, 6 months of CMR imaging is recommended (Fig. 5).

### Specialty CMR

**Fig. 6:**
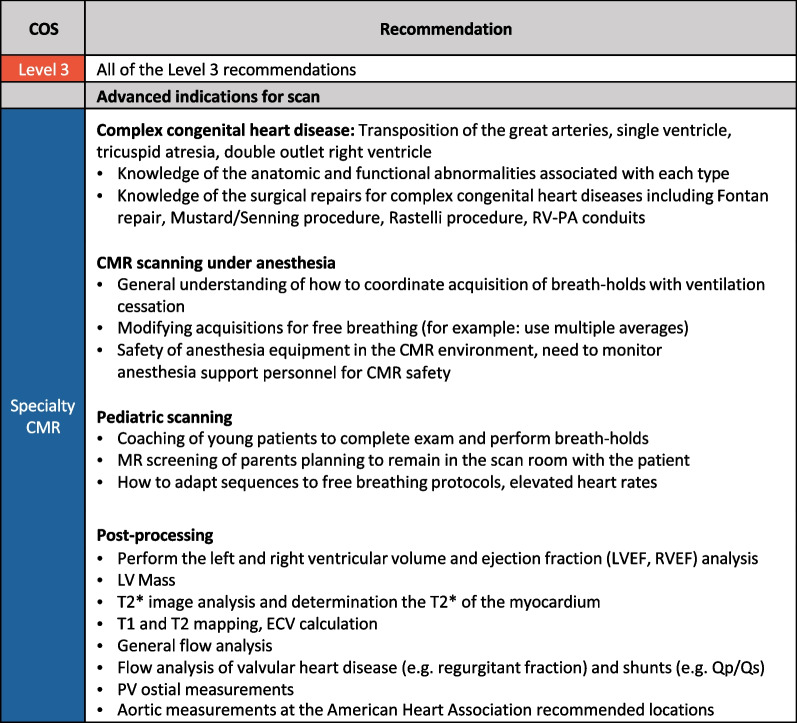
Specialty CMR skills. *ECV* extracellular volume fraction, *LVEF* left ventricular ejection fraction, *Qp/Qs* pulmonary to systemic flow ratio, *PA* pulmonary artery, *RVEF* right ventricular ejection fraction

Specialty CMR refers to procedures that are not routinely performed at many CMR centers. These include centers that perform examinations for complex CHD, pediatric CMR imaging, CMR scanning under anesthesia and CMR post-processing. In addition to all the Level 2 and Level 3 CMR requirements, it is recommended that the technologist has performed the following number of scans: Complex CHD-50 exams, Pediatric CMR-30 exams, CMR scanning under anesthesia-10 exams, CMR post-processing-20 exams (Fig. 6).

### Continuing education and maintenance of competence

CMR is experiencing a rapid pace of pulse sequence and cardiac protocol evolution mandating that CMR technologists performing CMR exams be engaged in efforts to maintain their CMR skills. As part of the continuing education (CE) credits required to maintain technologist accreditation and/or foster knowledge of CMR advancements, a technologist should ensure that ≥ 25% of those CE credits are related to CMR for those at skill Level 1 and ≥ 50% for higher skill levels.

Performance of at least 100 CMR exams every 2 years is required for maintenance of Level 2 competence. Performance of at least 200 CMR exams every 2 years is required for maintenance of Level 3 competence.

It is also recommended that CMR technologists at or higher than Level 2 attend a local, regional, national or international meeting that includes CMR content every 1–2 years. Attendance (in person or virtual) at CMR focused meetings will foster education in a didactic format, encourage networking amongst CMR technologists, and provide access to cutting edge research and technical developments.

## Data Availability

Not applicable.

## Abbreviations

ARRT: American Registry of Radiologic Technologists
CE: Continuing education
CHD: Congenital heart disease
CMR: Cardiovascular magnetic resonance
ECG: Electrocardiogram
MR: Magnetic resonance
MRA: Magnetic resonance angiography
SCMR: Society for Cardiovascular Magnetic Resonance
